# Evidence of Public Engagement with Science: Visitor Learning at a Zoo-Housed Primate Research Centre

**DOI:** 10.1371/journal.pone.0044680

**Published:** 2012-09-13

**Authors:** Bridget M. Waller, Kate Peirce, Heidi Mitchell, Jerome Micheletta

**Affiliations:** 1 Centre for Comparative and Evolutionary Psychology, Department of Psychology, University of Portsmouth, Portsmouth, United Kingdom; 2 Marwell Wildlife, Winchester, United Kingdom; Université Paris 13, France

## Abstract

Primate behavioural and cognitive research is increasingly conducted on direct public view in zoo settings. The potential of such facilities for public engagement with science is often heralded, but evidence of tangible, positive effects on public understanding is rare. Here, the effect of a new zoo-based primate research centre on visitor behaviour, learning and attitudes was assessed using a quasi-experimental design. Zoo visitors approached the primate research centre more often when a scientist was present and working with the primates, and reported greater awareness of primates (including conservation) compared to when the scientist was not present. Visitors also reported greater perceived learning when the scientist was present. Installation of information signage had no main effect on visitor attitudes or learning. Visitors who interacted with the signage, however, demonstrated increased knowledge and understanding when asked about the specific information present on the signs (which was related to the ongoing facial expression research at the research centre). The findings show that primate behaviour research centres on public view can have a demonstrable and beneficial effect on public understanding of science.

## Introduction

Primate cognitive and behavioural research is increasingly undertaken on public view in zoos and wildlife parks. Such research has the potential to engage the public with science as it happens long before findings are published and disseminated for public consumption. The research could be making a direct and positive impact on society by influencing attitudes to science and/or providing education about specific topics. Demonstrating a tangible impact on society is an important goal for scientists who are often required to evidence the wider reach of their research [1]. Impacting on the public in this way is often termed public engagement with science. Evidencing public engagement with science is challenging, however, and studies have had mixed success.

Most studies have assessed zoo visitor engagement by monitoring how visitors move through exhibits [1], [2], [3]. For example, visitor ‘dwell time’ (time spent at a specific site around the exhibit) is often used as a measure of engagement [1]. If visitors spend longer at an exhibit, this is taken to demonstrate that they are interested in and attending to the exhibit. A recent study used dwell times to assess how visitors were engaging with a new primate research centre at Edinburgh Zoo (Living Links to Human Evolution Research Centre: Living Links) [1]. Visitors showed substantial dwell times at the exhibit (reported as high relative to zoo standards), which can be interpreted as a measure of successful engagement [1]. The factor that affected visitor attention to the greatest extent was presence of a scientist (in real time) working with the primates. Additional signage and interactive materials installed in the exhibit significantly increased the dwell time of those visitors who interacted with materials, and overall dwell time in the exhibit increased after installation. Approximately two thirds of visitors engaged with the signage in some way at Living Links, but other studies have reported lower proportions. In an immersive exhibit in Lincoln Park Zoo [2] the majority of visitors (over 90%) did not read information signs or engage with the materials at all. Other studies have shown that materials with ‘hands on’ aspects are used more frequently than materials that require passive viewing [3]. On the whole, however, zoo visitors tend to spend more time watching the animals than reading signs [3], which is not unexpected given that seeing animals is likely to be a strong motivation for visiting the zoo. Nevertheless, given that zoos attract very large numbers of visitors (Lincoln Park has 3 million visitors annually [2]), engaging even a small proportion of this figure with educational materials could have a significant effect on public understanding of science. On the whole, these studies highlight that zoos have the potential to educate a large number of people.

Although increased time spent at an exhibit is highly suggestive of greater potential for educational impact [4], it is important to demonstrate that interest is positively associated with accurate knowledge transfer and learning. Portrayal of primates in entertainment media (such as adverts and films) has been suggested to adversely affect public perception of their conservation status [5]. Similarly, observers judge species to be less endangered if they see them in photographs in the presence of a human, and also consider them to make better pets [6]. Importantly, species seen in anthropomorphic settings (such as an office) were also judged to have more stable wild populations. Thus, it is possible that seeing primates interacting with human scientists could have a negative effect on public understanding of primate science and conservation, rather than the desired positive effect. Animal training demonstrations in zoos, however, *have* been shown to have a positive effect on visitor learning [7], [8], but we cannot assume this is true of live scientific demonstrations with primates without empirical evidence.

The current study assessed the impact of a new zoo-based primate research centre (The Macaque Study Centre at Marwell Wildlife, Hampshire, UK) on public engagement and visitor learning, using observations of visitor behaviour and questionnaires.

## Materials and Methods

### The Macaque Study Centre

The Macaque Study Centre is a newly built facility for cognitive and behavioural research with crested macaques (*Macaca nigra*) at Marwell Wildlife Zoological Park, in Hampshire, England (with approximate visitor numbers of 400,000 per annum). Funded by the University of Portsmouth (UoP), the facility represents a collaborative venture between UoP and Marwell Wildlife to conduct high quality research and engage the public in ongoing scientific work. The main scientific goals are to investigate crested macaque social cognition and behaviour (e.g. [9]) with a specific focus on facial expression [10].

**Figure 1:**
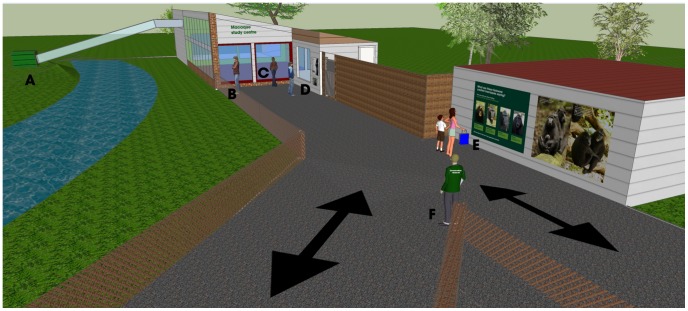
Overview of the Macaque Study Centre. A) Macaque island, B) viewing point to the macaque island, C) macaque inside enclosure, D) research area where the scientist can be seen working with the macaques, E) facial expression sign with interactive flaps, and F) the researcher collecting observational and questionnaire data.

The Macaque Study Centre is a small building extension to the existing macaque enclosure, consisting of a testing room (designed for the macaques to enter) and a research area (for the scientists) (see Figure 1). The macaques can voluntarily enter the testing room from their enclosure, and interact with the scientists through a mesh and toughened plastic interface. The macaques are trained to participate in experimental tasks using computerised touchscreens. Visitors to the zoo can observe both the scientist and the macaque through a large window into the research area (Figure S1), but they cannot interact with the scientist. Visitors can also see the macaques operating the touchscreen on a monitor showing live footage from the testing room. Research sessions took place three days a week (for approximately 2 hours, varying between 10am and 4pm), so there were times when the scientist was not present.

### Information signage

**Figure 2:**
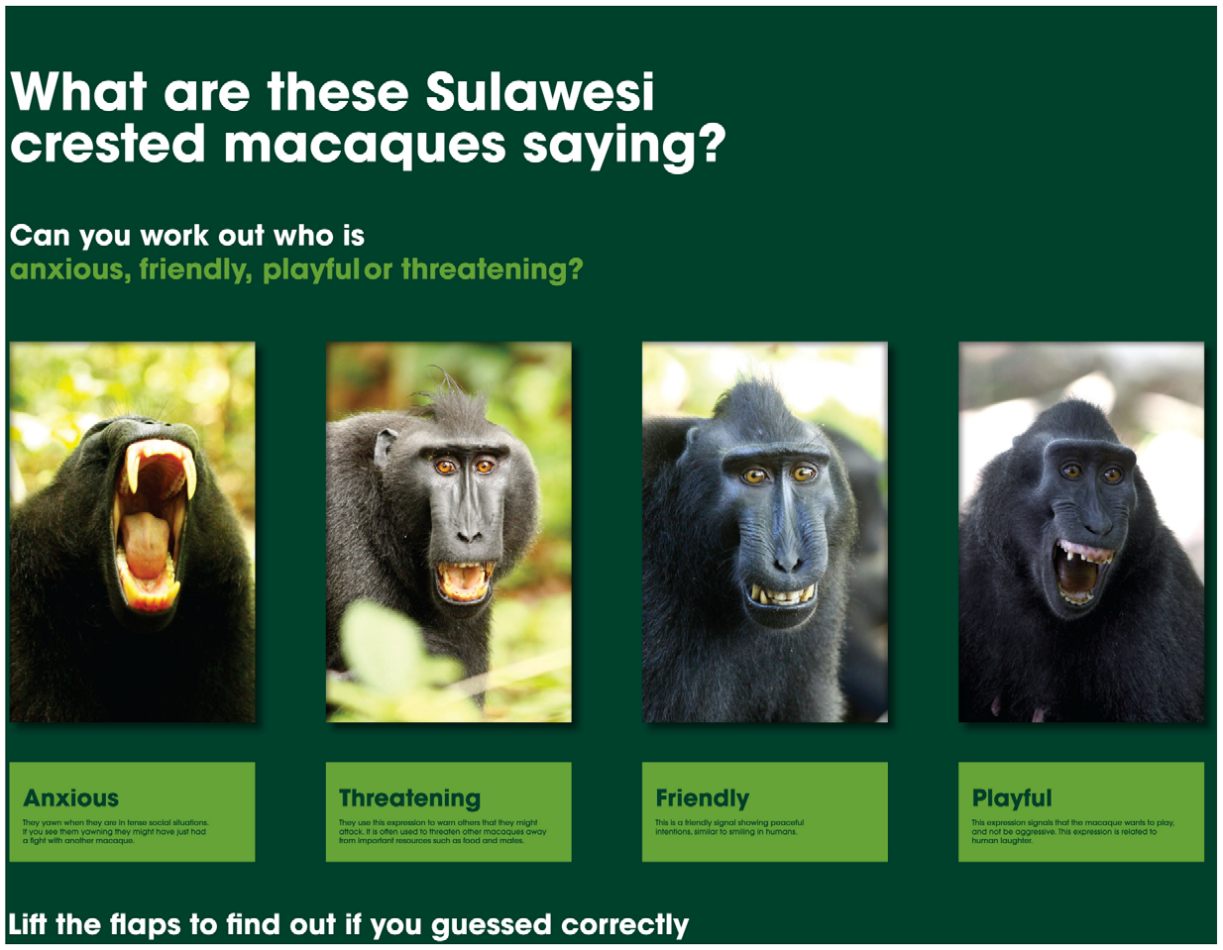
Facial expression sign with interactive materials (flaps cover the descriptions and can be lifted by visitors).

In November 2011, information signage was installed around the Macaque Study Centre (Figure S2). The majority of panels consisted of large photographs of crested macaques, with brief textual information about their behaviour and social organisation (e.g. dominance hierarchy, maternal care, group living), and one panel including more detailed text about the overarching scientific project. One interactive panel was also installed, consisting of 4 facial expression photographs and descriptions about social function and meaning. The descriptions are all hidden under flaps that can be lifted by zoo visitors, enabling them to guess what facial expressions mean, before seeing the answers under the flaps (Figure 2).

### Participants

One hundred and fifty five visitors (89 female) to Marwell Wildlife were recruited for participation, aged 16–84 years (M = 37.49, SD = 11.48), between September 2011 and March 2012. One hundred and twenty visitors had visited before, and 23 were regular visitors (annual pass holders).

### Design

A between-subjects quasi-experimental design was used to test the effect of two independent variables (presence of scientist and presence of information signage), each with two levels (presence and absence), on dependent variables of visitor attitudes, perceived visitor learning and actual visitor learning (using a face to face questionnaire). Eighty participants were in the pre-signage condition, 76 in the signage condition, 77 in the scientist present condition, and 79 in the scientist not present condition. Participants in the signage condition were split into two further conditions (post-hoc) depending on whether they interacted with the signage. Of the 76 in the signage condition, 30 interacted with the signage.

### Observational procedure

Visitors to Marwell Wildlife were observed as they entered the Macaque Study Centre area (see Figure 1). One/zero sampling [11] was used to record their behaviour as they navigated the area. We recorded whether they 1) approached the viewing point to the macaque island, 2) approached the macaque enclosure window, and 3) approached the viewing window to the Macaque Study Centre research area. Approaching was defined as walking to (and pausing in) a defined area (regardless of whether they stayed in the area for any length of time), and was recorded in real time. Observers were trained until they reached 100% reliability with the main observer (KP).

As a measure of engagement with the signage, we recorded whether visitors lifted the flaps on the interactive facial expression sign (Figure 2). Figure 2 shows four crested macaque facial expressions with a description of their social context and communicative function hidden under a flap. Visitors are invited to guess the ‘meaning’ of each facial expression, and check by lifting the flap. We used this behaviour (lifting the flaps) to assign participants in the signage condition to one of two further conditions. Those who physically lifted the flap were deemed to have more fully engaged and interacted with the signs than those who did not.

As they left the area (Figure 1), visitors were approached and asked if they would like to participate in a study by answering a short questionnaire about their experience at the zoo. If they agreed, they were given an informed consent form to sign, and the questionnaire to be completed.

### Questionnaire

If visitors agreed to take part, they were asked to complete an individual 9-item questionnaire (Questions A1–A9, see Table 1) about their attitudes to science and primates, and their perceived learning experience at the Macaque Study Centre. Responses were recorded on 7-point Likert scales with anchors appropriate to the questions (e.g. Question A1: “Not at all interested” to “Extremely interested”.

**Table 1:** Individual scores of the principal components analysis (PCA) performed on the visitor responses.

Question	Component		
	1 (2.14)‘agreement’	2 (1.31)‘perceived learning’	3 (1.00)‘awareness’
A1: How interested are you in primates?	0.23	0.01	**0.66**
A2: How important is it to save primates from extinction?	**-**	-	-
A3: How important is it to conduct scientific research in zoos?	**0.85**	0.17	0.11
A4: Do you think we can learn much from studying primates?	-	-	-
A5: Do you think scientific research in zoos is good for the animals?	**0.85**	0.09	0.06
A6: Have you learnt anything about primates during your visit today?	0.11	**0.81**	0.17
A7: Have you learnt anything about science during your visit today?	0.14	**0.81**	0.01
A8: Do you think crested macaques are in danger of becoming extinct?	0.10	0.04	**0.74**
A9: Do you think that primates think like we do?	−0.26	0.32	**0.61**

Note. The highest loading is in bold and shows which component the question is assigned to. Eigenvalues for each component are given in parentheses.

If visitors were in the signage condition, they were asked an additional 3 questions designed to assess actual learning, rather than perceived learning (see Table 2, Questions B1–B3). The questions asked about information given on the facial expression sign (Figure 2), and related to the ongoing scientific research being conducted at the Macaque Study Centre (primate communication research). Visitors were asked a broad question about evolutionary theory ‘Do you think we share ancestors with crested macaques’ and a specific topic based question ‘Do you think yawning shows that primates are relaxed?’. For the latter, they responded on a 7-point Likert scale with anchors from 1 “Not at all” to 7 “Completely”. The interactive facial expression sign showed a crested macaque yawning face, and hidden under a flap the description “Anxious: They yawn when they are in tense social situations. If you see them yawning they might have just had a fight with another macaque”. Thus, we expected visitors to make a lay assumption that yawning indicates tiredness, but to answer differently if they had read the sign. After reading the sign, we expected them to understand that yawning individuals are not necessarily tired, but are in fact less likely to be relaxed than other individuals [12], [13].

**Table 2:** Visitor responses to the individual questions.

Question	Mean responses (out of 7+SD)					
	Exp. not present	Exp. present	Pre-signage	With signage		
					No interaction	Signage interaction
A1: How interested are you in primates?	5.16 (0.97)	5.29 (1.06)	5.35 (1.01)	5.09 (1.02)		
A2: How important is it to save primates from extinction?*	6.77 (0.60)	6.70 (0.76)	6.78 (0.50)	6.68 (0.84)		
A3: How important is it to conduct scientific research in zoos?	6.05 (0.10)	5.87 (1.20)	6.11 (1.03)	5.82 (1.16)		
A4: Do you think we can learn much from studying primates?*	6.23 (0.83)	6.20 (1.11)	6.28 (1.01)	6.16 (0.95)		
A5: Do you think scientific research in zoos is good for the animals?	5.34 (1.18)	5.47 (1.39)	5.54 (1.23)	5.26 (1.34)		
A6: Have you learnt anything about primates during your visit today?	4.18 (1.44)	4.63 (1.62)	4.61 (1.55)	4.20 (1.52)		
A7: Have you learnt anything about science during your visit today?	3.62 (1.50)	4.15 (1.71)	4.05 (1.64)	3.72 (1.60)		
A8: Do you think crested macaques are in danger of becoming extinct?	4.91 (0.95)	5.41 (1.16)	5.06 (1.12)	5.26 (1.05)		
A9: Do you think that primates think like we do?	4.78 (1.35)	5.32 (1.20)	5.08 (1.48)	5.03 (1.10)		
B1: How much do you think primates communicate with each other?*	6.56 (0.67)	6.56 (0.70)			6.55 (0.72)	6.57 (0.63)
B2: Do you think we share ancestors with crested macaques?	5.44 (1.23)	5.02 (1.74)			5.09 (1.58)	5.47 (1.38)
B3: Do you think yawning shows that primates are relaxed?	3.85 (1.57)	4.05 (1.77)			4.37 (1.34)	3.20 (1.95)

* Questions were omitted due to mean responses above 6.0 (and thus interpreted as showing ceiling effects).

### Data analyses

All questions showing skewness or kurtosis +/−2 and/or ceiling effects (mean response >6.0 when the maximum response was 7) were removed from the analyses. Factor scores were normally distributed (tested using Kolmogorov-Smirnov test). Parametric analyses were used throughout.

### Ethics statement

Participation of visitors was entirely voluntary and written informed consent was gained prior to completing the questionnaire. A debriefing sheet was provided after participation. Observational data from visitors who had been observed (but who did not give informed consent) were not used. The procedures have been scrutinised and approved by the University of Portsmouth regulated Department of Psychology Ethics Committee.

## Results

### Descriptive statistics

Visitors in the two scientist conditions (scientist present or not) did not differ in age (t = 0.08, df = 150, p = 0.94) or sex composition (χ^2^(1) = 1.64, N = 154, p = 0.20). Visitors in the two signage conditions (signage installed or not) differed significantly in age (t = 2.03, df = 150, p<0.05), but as the means were close and in the same age category of the late thirties (signage not installed: 35.6; signage installed: 39.3) and standard deviations were very similar in both conditions (11.9 and 10.8 respectively) this was unlikely to influence responses in the two conditions. Sex composition did not differ between the two signage conditions (χ^2^(1) = 0.19, N = 154, p = 0.66). More visitors were sampled on weekends than weekdays after the signage was installed (compared to before installation), χ^2^(1) = 10.70, N = 155, p<0.05. However, visitors did not differ in their questionnaire responses on weekends compared to weekdays (agreement: t = −0.12, df = 153, p = 0.30; perceived learning: t = −0.32, df = 153, p = 0.75; awareness: t = 0.39, df = 153, p = 0.70). Visitors who lifted the flaps did not differ from those that did not in age (t = 1.68, df = 73, p = 0.10) or sex composition (χ^2^(1) = 0.33, N = 75, p = 0.64). Visitors who lifted the flaps did not differ from others in their reported interest in primates (Q1: t = −0.74, df = 74, p = 0.15).

### Questionnaire Responses: Principal Components Analysis

**Figure 3:**
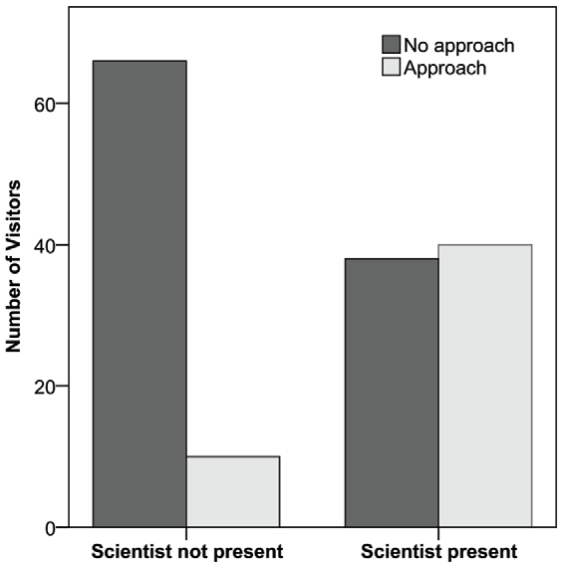
The number of visitors who approached the research window of the Macaque Study Centre in two conditions (scientist present and not present).

Questions A2 (‘How important is it to save primates from extinction?’), A4 (‘Do you think we can learn much from studying primates?’) and B1 (How much do you think primates communicate with each other?’ were removed from analysis due to ceiling effects (mean participant response >6.0). The questionnaire responses to the 9 questions relating to attitudes and perceived learning experience were subject to principal components analysis (PCA) with varimax rotation. PCA is a descriptive procedure that can be used to group questionnaire responses into related clusters, thus identifying any important underlying structure to how participant's respond (the principal components). The responses were reduced to three components accounting for 31.1%, 19.0% and 14.1% of the variance respectively. Items (questions) were included in the component in which they had the highest loading. The Kaiser-Meyer-Olkin measure of sampling adequacy was 0.63, and Bartlett's test of sphericity was significant (χ_2_ = 148.2, *p*<0.005), indicating that the data were appropriate for PCA. Table 1 shows the individual loading values for the questions. Questions included in Component 1 appeared to relate to *agreement with zoo-based research* (e.g., question A5 “Do you think scientific research in zoos is good for the animals?”). Component 2 seemed to relate to *perceived learning* (e.g., question A7 “Have you learnt anything about science today?”) and Component 3 seemed to relate to *interest and awareness of primates* (e.g., question A8 “Do you think crested macaques are in danger of becoming extinct?”). Figure 3 shows a 3D representation of the questions in relation to the axes (components). Reliability analyses were conducted to see how well the question responses were related to each other within each component, which gives an indication of the strength of the underlying structure of that component. Components 1 and 2 had moderate to high reliability (*agreement with zoo-based research*: Cronbach's α = 0.74; *perceived learning*: Cronbach's α = 0.60) but Factor 3 (*awareness*) had lower reliability (Cronbach's α = 0.44) indicating that the questions were not as well related. See Table 2 for the mean responses to individual questions.

### Repeat Visitors

Visitors who had visited before did not exhibit different responses to those who were visiting for the first time, on any of the questionnaire components (*agreement*: t = −1.30, df = 152, p = 0.20; *perceived learning*: t = 1.12, df = 152, p = 0.26; *awareness*: t = −1.56, df = 152, p = 0.12).

### Presence of Scientist

**Figure 4:**
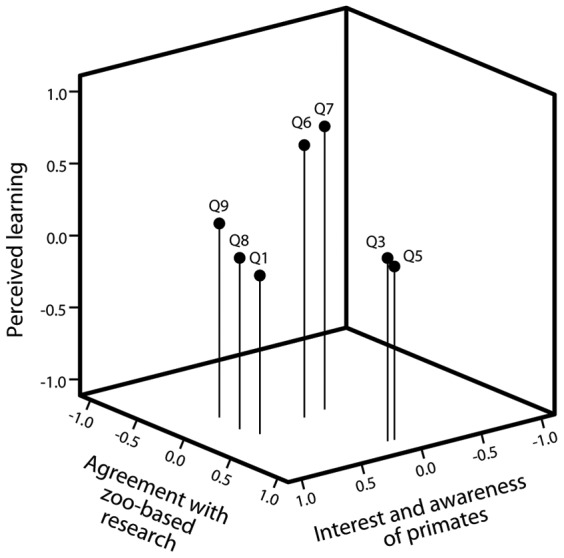
Loading of the individual questions (see **table 2** for full questions) on each component derived from the principal components analysis (PCA).

There was a significant association between presence of scientist, and whether visitors approached the Macaque Study Centre, χ^2^(1) = 25.52, N = 154, p<0.001 (see Figure 4). Visitors were 6.9 times more likely to approach the research window of the centre if the scientist was present (based on the odds ratio): 51% approached when the scientist was present, compared to 13% who approached when the scientist was not present. There was no association between presence of scientist and approaching the macaque island, χ^2^(1) = 0.52, N = 154, p = 0.469, or between presence of scientist and approaching the macaque enclosure window, χ^2^(1) = 0.72, N = 154, p = 0.396.

**Figure 5:**
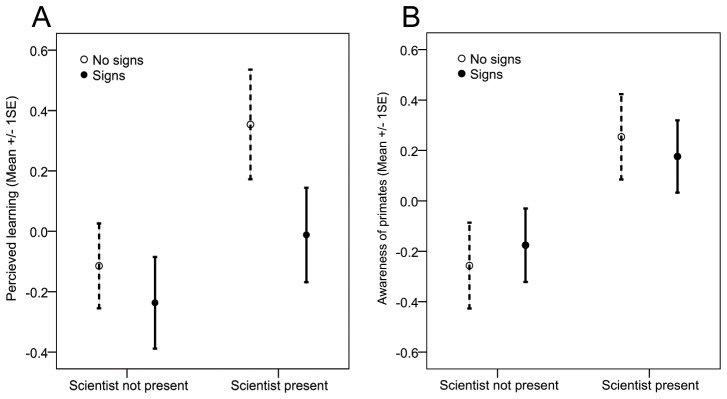
a) Visitor perceived learning when scientist was present (or not) and before and after signage installation, b) Visitor awareness of primates when scientist was present (or not) and before and after signage installation.

The 3 components identified by the PCA were used as dependent variables in a 2 (pre-signage vs post-signage)×2 (scientist present vs not present) between subjects ANOVA. There was a significant positive main effect of scientist presence on *perceived learning*, F(1,151) = 4.79, p<0.05, η^2^ = 0.03 (see Figure 5a), and *awareness of primates*, F(1,151) = 7.43, p<0.05, η^2^ = 0.05 (see Figure 5b), but not on *agreement with zoo-based research*, F(1,151) = 0.42, p = 0.42, η^2^ = 0.004. All effect sizes were small, demonstrating that only a small amount of the variance is explained by the difference in conditions (which is not unexpected given the many variables affecting human attitudes).

### Presence of Signage

There were no main effects of signage and no interaction effects of scientist and signage.

### Engaging with Signage

Of the 76 visitors in the signage present condition, 30 (39%) lifted the flaps and were thus deemed to have engaged with the signs more than those who did not lift the flaps. Visitors were thus assigned to one of two conditions (engaged with signage vs did not engage with signage). Responses to the additional knowledge assessment questions (those related to information present on the signs) were entered into 2 (scientist present vs not present)×2 (engaged with signage vs did not engage with signage) between subjects ANOVA. There was a main effect of engaging with the signage on the question relating to yawning (B3: “Do you think yawning shows that primates are relaxed?”), F(1,72) = 10.86, p<0.005, μ^2^ = 0.13. Participants who engaged with the signage thought that primates are less relaxed when they yawn, than those who did not engage with the signage. There was no main effect of engaging with the signage on question B2 (“Do you think we share ancestors with crested macaques?”), F(1,72) = 0.85, p = 0.359, μ^2^ = 0.01. There was no main effect of presence of scientist, and no significant interactions.

## Discussion

Zoo visitor's perceived learning, and interest and awareness of primates were increased when a scientist was present and the visitors could watch the scientist interact with the animals. We found no effect of the signage installation on overall mean visitor attitudes or learning, either independently or when the scientist was present. Those visitors who more fully attended to and engaged with the signs, however, (identified as those who physically lifted the interactive flaps on one sign) demonstrated *actual* learning when they were asked about specific information present on the signs. As the proportion of visitors who engage with signage is relatively small [2], any effect of the signs might be obscured by the fact that so few visitors read them. Focussing on those visitors who did engage by isolating those who lifted the flaps, however, shows that visitors *can* learn from signage. Indeed, if 39% of total visitors to the zoo lifted the flaps, that could translate to very large numbers of people learning from the exhibit. On the whole, the results are encouraging and demonstrate that primate behaviour research centres on public view can have a positive, tangible effect on public education. The findings suggest, however, that some aspects of such facilities could be more effective than others at communicating science.

Many more visitors approached the research window of the Macaque Study Centre when a scientist was present, showing that the building itself (and internal equipment like the touchscreen) did not attract visitor attention. Visitors who had the opportunity to watch a scientist work with the macaques reported greater awareness of primates, than those who did not. Visitor responses that were most affected by presence of the scientist were those relating to whether the primates ‘think like we do’ (perhaps unsurprising given that the crested macaques were observed engaging in cognitive tasks) and those relating to the conservation status of the species. Specifically, those visitors who had the opportunity to watch the scientist reported that crested macaques are in greater danger of becoming extinct. These visitors may have reflected on conservation issues as they had more opportunity to observe the animals, and as we did not record dwell times, it is possible that this effect is influenced by increased time spent watching the animals. Further studies are needed to tease apart these explanations. Alternatively, they may have also absorbed more information from signage (although there was no interaction effect with the signage installation, so this is unlikely). Nevertheless, this is a very encouraging finding given that perception of conservation status can be adversely affected by seeing primates in human settings [5], [6], which does not seem to have been a factor here. It is also encouraging that the question relating to how important it is to save primates from extinction received ceiling responses, and had to be removed from analysis (the mean response to the question was near the top of the scale, showing that the vast majority of the visitors had good awareness of the need for conservation).

Visitors who watched the scientist at work reported greater perceived learning effects than those who did not see the scientist. Visitors also reported that they had learnt more about science during their visit. This is an encouraging finding and supports previous research showing greater perceived learning when visitors watch zoo animals being trained [8], [14]. Visitor's also demonstrated learning when they interacted with the signage. Installation of the signage alone had no effect on visitor attitudes or learning, which supports previous research that very few visitors look at signage [1], [3]. Yet when visitors fully engaged with the signage (lifting the flap taken as a measure of paying more attention to the sign), and were then asked about the specific information they had read under the flap (which was, perhaps, counter to their intuitive, lay impressions about facial expressions), they demonstrated an increase in knowledge and understanding. This supports previous research which suggests that there may be differential effects of exhibits on visitors depending on whether they engage with educational materials at all [1]. Visitors who had better attended to the sign about facial expression had better understanding about primate yawning, and what it communicates to others, than if they had not fully attended to the sign. This finding is particularly encouraging as the scientific information relates precisely to work being conducted at the research facility (facial expression research), suggesting that very specific scientific dissemination could be occurring. Also, as the question required the visitor to reflect on information they had read (and not simply produce verbatim, arbitrary recall) this could even be interpreted as meaningful learning [15]. Interestingly, when asked about more general information relating to the same sign (“How much do you think primates communicate with each other?”), they did not respond differently to those visitors who had not lifted the flaps. Future studies are needed to determine whether this is due to different types of information being more easily transmitted by this form of engagement, whether specific aspects of the sign are important, and whether this knowledge is retained long-term.

It is possible that the visitors that interacted with the signage differed in some way from those who didn't, although we found no significant differences in age, sex or interest in primates. It is also possible that the visitors sampled before and after signage installation differed in some way, especially as they were sampled during different seasons and visitor behaviour is likely to differ when rainfall and temperature differs. The results relating to the signage should therefore be taken with caution. Finally, as we sampled only those visitors who consented to take part in the study, it is likely that they had greater interest and knowledge in the topic than those visitors who did not want to take part. In sum, our sample may not be generalisable to all zoo visitors.

### Conclusion

Primate behaviour research is increasingly occurring on public view in zoo settings, as scientists, funders and zoos are becoming more aware of the excellent potential for public engagement with science. Evidence of specific knowledge transfer, learning, and attitudinal change, however, has been elusive. Here, we provide quantitative evidence that zoo visitors can be positively affected by visiting a primate behaviour research centre on public view in a zoo setting. Zoo visitors approached the primate research centre more often when a scientist was present and working with the animals, showing that live demonstrations assisted the public in engaging with the science. Importantly, however, we have also demonstrated that zoo visitors gained something from this experience. Zoo visitors exhibited increased awareness of the conservation status of crested macaques and reported a greater perceived learning experience when observing scientists at work with the animals. Zoo visitors also demonstrated an increase in knowledge and understanding if they interacted with information signage relating to specific topics relevant to the scientific research. Overall, the findings suggest that primate behaviour research centres hold enormous potential for public engagement with science.

## Supporting Information

Figure S1 **Visitor window to the research area where the scientist works with the macaques.** (Note. The subject of the photograph has given written informed consent, as outlined in the PLoS consent form, to publication of their photograph.)
(TIF)

Figure S2 **Information signage installed in various locations around the Macaque Study Centre.**
(TIF)
