# Supplementary figures and images for: Evidence of Public Engagement with Science: Visitor Learning at a Zoo-Housed Primate Research Centre
Source: PLoS One. 2012 Sep 13;7(9):e44680. doi: 10.1371/journal.pone.0044680 (PMC3441616; doi:10.1371/journal.pone.0044680)

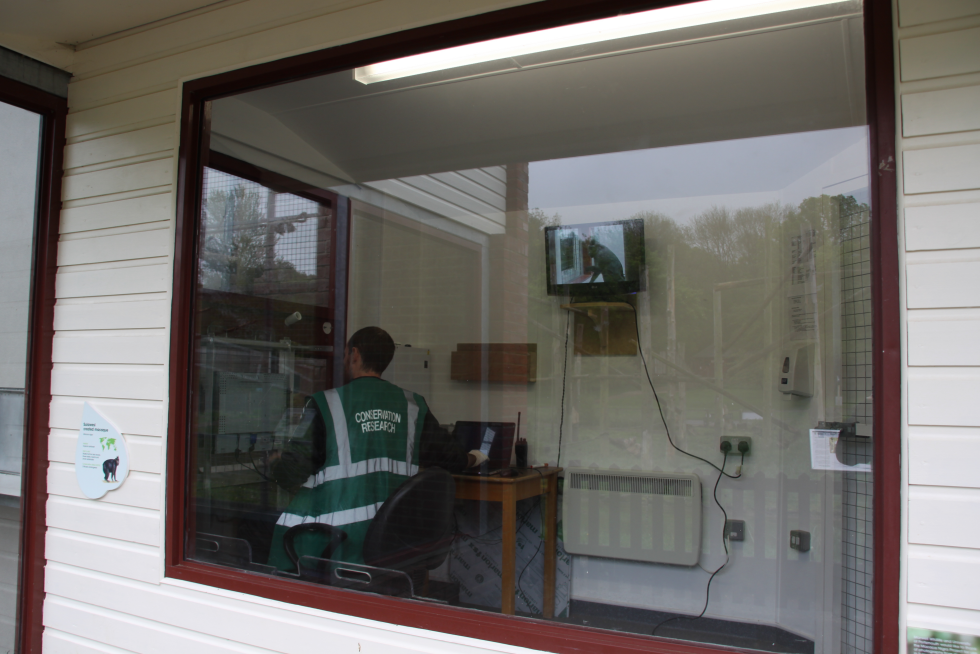

Supplement: Figure S1 — Visitor window to the research area where the scientist works with the macaques. (Note. The subject of the photograph has given written informed consent, as outlined in the PLoS consent form, to publication of their photograph.) (TIF) [file pone.0044680.s001.tif]

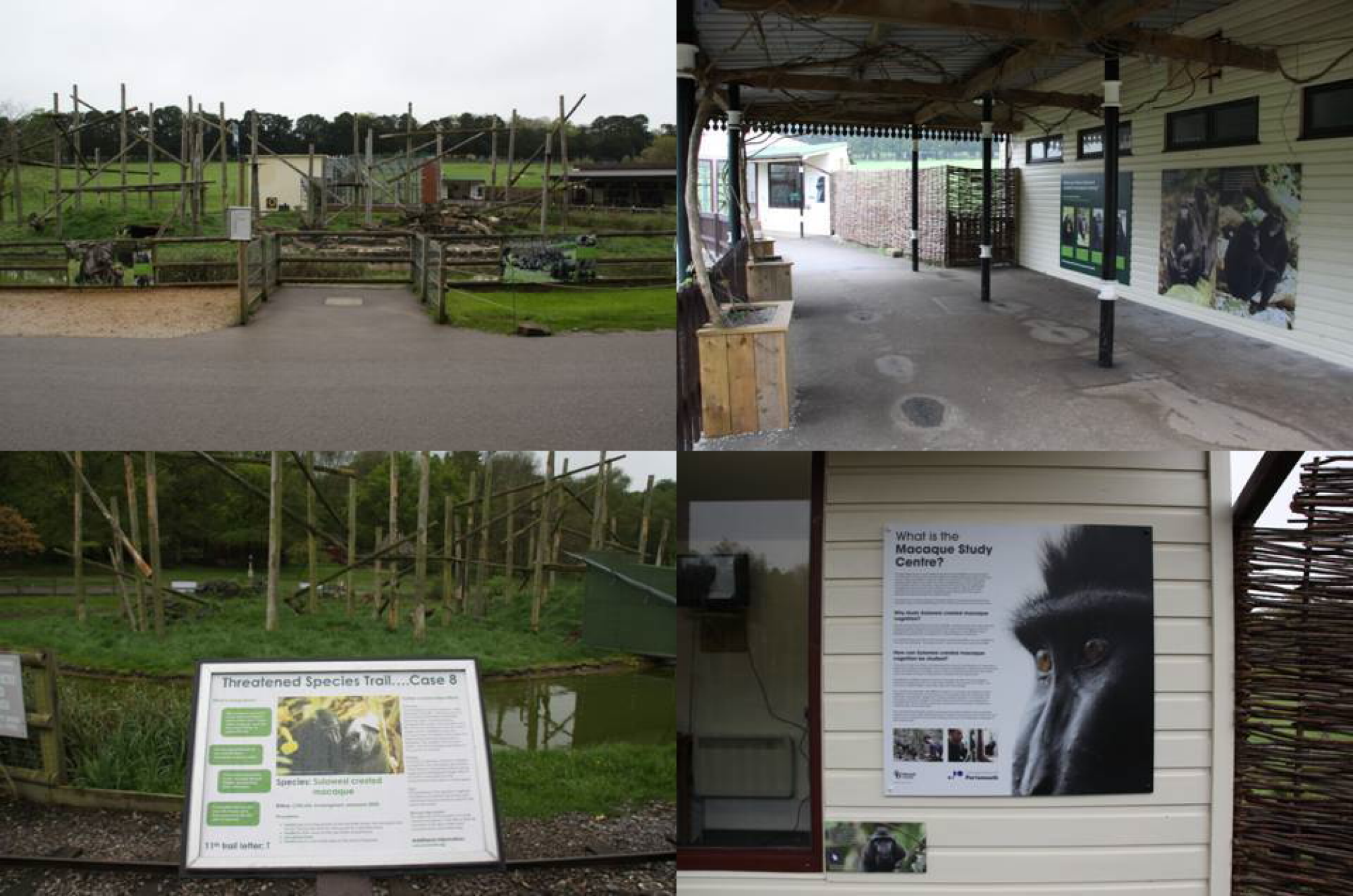

Supplement: Figure S2 — Information signage installed in various locations around the Macaque Study Centre. (TIF) [file pone.0044680.s002.tif]
